# Psychometric properties and qualitative evaluation of a Swedish translation of the New Sexual Satisfaction Scale–Short (NSSS-S)

**DOI:** 10.1371/journal.pone.0330353

**Published:** 2025-08-25

**Authors:** Amanda Klysing, Ingela Steij Stålbrand, Laura del Carmen Sánchez-Sánchez, Tove Lundberg

**Affiliations:** 1 Department of Psychology, Lund university, Lund, Sweden; 2 Department of Personality, Evaluation and Psychological Treatment, University of Granada, Granada, Spain; University of Perugia: Universita degli Studi di Perugia, ITALY

## Abstract

**Objectives:**

Sexual satisfaction is a crucial part of sexual health and is positively related to well-being and quality of life. However, there are at present no composite measures of sexual satisfaction available in Swedish. The New Sexual Satisfaction Scale – Short form (NSSS-S) is a measure of sexual satisfaction that has been validated in multiple languages, and the aim of the present study is to evaluate the psychometric properties and intelligibility of a Swedish version of the NSSS-S.

**Methods:**

A convenience sample (*N* = 479) responded to an online survey including the Swedish NSSS-S and a single-item measure of sexual satisfaction. Qualitative evaluation of the translation was done within 20 semi-structured interviews where respondents gave open answers to the NSSS-S items.

**Results:**

Results showed that a two-factor solution, matching the subscales Ego-Centred and Partner/Activity-Centred sexual satisfaction, showed good to acceptable fit to the data. However, removal of the item “Partner’s ability to orgasm” increased model fit significantly. Internal reliability for the full scale was high and NSSS-S scores correlated strongly with the single-item measure of sexual satisfaction. Qualitative evaluation showed no issues with comprehension of the translated items or with the semantic and cultural validity of the translated measure, but revealed additional aspects of measuring sexual satisfaction not captured by a scale such as the NSSS-S.

**Conclusions:**

The Swedish NSSS-S displayed the expected bi-dimensional factor structure, high convergent validity in relation to the other available measure of sexual satisfaction and can be used with both partnered and single respondents. Qualitative evaluation indicated that the NSSS-S is generally seen to capture important elements of measuring sexual satisfaction but that it may not adequately capture experiences of situational and temporal variability in sexual satisfaction. The Swedish NSSS-S thus showed comparable psychometric and semantic properties to the original version and can be used to measure sexual satisfaction in a Swedish speaking population.

## Introduction

Sexual health means having a positive and respectful attitude towards sexuality as well as the ability to have pleasurable and safe sexual experiences [1]. One important part of sexual health is sexual satisfaction [2], which is often defined as the affective response following a person’s subjective assessment of their sexuality [3], and is dependent on physiological, psychological, interpersonal and societal/cultural factors [4,5]. Higher sexual satisfaction generally results in better subjective wellbeing [6] and a better quality of life [7] and is thus an important aspect of public health [8], as well as a common target of therapeutic interventions (e.g., [9]).

In the most recent public sexual health survey in Sweden, results showed large inequalities in sexual satisfaction within the population [10]. For example, only 32% of men aged 16–29 were satisfied with their sex life in the past year and as many as 30% of women aged 30–44 lacked sexual desire. These inequalities of sexual satisfaction in the population are assumed to be caused by structural factors relating to age, gender and level of education and individual factors such as empowerment, self-esteem, and integrity [10]. For instance, age and education level have been found to be negatively correlated with sexual satisfaction [11], while sexual assertiveness and sexual self-esteem are positively correlated with sexual satisfaction [12]. Drawing on these facts, a national strategy for sexual and reproductive health has been issued by the Swedish Government which aims to promote a good and equal sexual health, and this includes decreasing sexual dissatisfaction in the population [13]. However, to provide a solid research basis for how to promote good and equal sexual health in Sweden, more knowledge is needed about how both structural and individual factors affect sexual satisfaction. This requires the use of conceptually sound and validated measures of sexual satisfaction. While some research has been done on sexual satisfaction in Sweden [14,15], implications of these studies are limited by the lack of thorough and validated measures of sexual satisfaction in Swedish. Due to this lacuna, most studies performed in Sweden have instead included single-item measurements of overall satisfaction with one’s sex life (e.g., [10,14]). Furthermore, available quantitative research conducted with Swedish participants on factors associated with sexual satisfaction is almost exclusively focused on clinical populations [15–19] making it difficult to assess the extent to which international research findings on sexual satisfaction among a general population are generalizable to a Swedish context. The most thoroughly evaluated measure of sexual satisfaction in Swedish is an item from the Life Satisfaction Scale which was developed to assess satisfaction with eight life domains, including satisfaction with sexual life [20]. Psychometric assessment of the Life Satisfaction Scale showed that the sexual satisfaction was a facet of a generally emotion-focused cluster of domains with satisfaction with partnership relations, family life, and ability to manage self-care needs [20]. This single-item measure has been included in several Swedish studies on sexual satisfaction [15,17,19] and has been shown to be a useful screening tool for sexual dysfunction [21]. Using a single item, rather than a composite scale, to measure a multi-faceted construct such as sexual satisfaction can, however, jeopardize validity through not capturing all the aspects included in the construct (see, e.g., [22]), and has an increased risk of low reliability of measurement [23]. Translating a theoretically and psychometrically sound composite measure of sexual satisfaction into Swedish would therefore greatly improve knowledge production regarding sexual satisfaction levels and predictors as well as make it possible to conduct cross-cultural research comparing Sweden to other cultures.

In a systematic review of research on sexual satisfaction from 1979–2012, Sánchez-Fuentes et al. [4] identified more than 40 different scales and items in use and concluded that 12.7% of studies used a single-item measure, 12.2% of studies used the Index of Sexual Satisfaction [24] and 9.6% used the unidimensional Global Measure of Sexual Satisfaction [3]. Further, this review identified a general dearth of theoretical grounding of measures of sexual satisfaction, with the GMSEX and, the more recently developed, bidimensional New Sexual Satisfaction Scale [2] being identified as the only two currently used measures that address both individual and interpersonal aspects of sexual satisfaction [5]. To directly test how different sexual satisfaction measures compare, Mark et al. [23] performed a psychometric comparison of the ISS [24], the GMSEX [3], the short form of NSSS [25], and a single-item measure of sexual satisfaction. The ISS failed to meet several of the psychometric criteria, the single-item measure performed moderately well in terms of convergent validity, and the GMSEX emerged as the preferred unidimensional measure of sexual satisfaction [23]. However, the NSSS-S performed comparably to the GMSEX in terms of psychometric properties with additional benefit of being able to differentiate between aspects of sexual satisfaction related to the self and aspects related to a sexual partner [23]. The NSSS-S is therefore suggested as a valid and reliable measure of sexual satisfaction as a bidimensional construct.

The NSSS was developed by examining the issues addressed in therapeutic interventions for sexual dysfunction, and items were chosen to allow for a systematic measurement of sexual satisfaction as related to relational, individual, and behavioural aspects [2]. Furthermore, the NSSS was developed to be applicable to a wider range of target groups compared to previous composite measures and serve as a measure of sexual satisfaction for respondents with varying sexual orientations, genders, and relationship statuses [2]. While the scale was conceptually designed with a separation between relational, individual, and behavioural aspects, initial dimension reduction showed that the scale items split into two subscales: one measuring Ego-Centred sexual satisfaction (individual aspects) and one measuring Partner- and Activity-Centred sexual satisfaction (relational and behavioural aspects). The NSSS was further developed into a 12-item short form (NSSS-S) to make the measure easier to implement in research and clinical settings [25]. The short version of NSSS shows good psychometric properties, including high internal consistency and adequate test-retest reliability, which are comparable to the long version [23,25]. Validated translations of the NSSS-S from English are available for Spanish [26], Portuguese [27], French [28], and German [29]. However, while some studies have shown that the NSSS-S displays the same two-dimensional structure as the NSSS does [23,27], others have instead found a one-dimensional factor structure [25,29]. This inconsistency points to the importance of thorough evaluation of the factor structure of versions of the NSSS-S, to specify if the scale can be used to test differences in Ego-Centred and Partner/Activity-Centred sexual satisfaction or should only be used as a general measure of sexual satisfaction.

The NSSS and the NSSS-S have been found to be useful tools in multiple languages to evaluate the relationship between sexual satisfaction and different aspects of well-being. Sexual satisfaction as measured by the NSSS or the NSSS-S is positively correlated with positive relationship aspects, including relationship intimacy, partner communication about sex [2], general relationship satisfaction [23,30], and general life satisfaction [2]. Further, NSSS or NSSS-S scores are negatively related to sexual boredom in Croatian, Portuguese, and US samples [2,27] as well as negatively related to perfectionism [31]. Providing a translated and validated Swedish version of the NSSS-S would therefore enable additional research in a Swedish context on how different structural, relational, and individual factors influence facets of sexual satisfaction.

The aim of the current study is to translate the NSSS-S into Swedish and evaluate its psychometric properties and intelligibility. Confirmatory factor analysis (CFA) is used to test if the internal structure of the Swedish NSSS-S fits a unidimensional factor structure or a bidimensional structure, multi-group CFAs are used to assess measurement invariance based on relationship status, and evidence for the validity of the Swedish NSSS-S based on relation to other measures is evaluated through the relation of scores on the Swedish NSSS-S to a single item-measure used in previous research on sexual satisfaction [20]. Finally, respondents’ understanding of the items is probed in a follow-up sample using short, semi-structured interviews. This mixed-methods approach addresses concerns regarding potential discrepancies between research and layperson definitions of sexual satisfaction [32] and follows recommendations for cultural adaptation of translated self-report health measures [33].

## Materials and methods

The current study consisted of two parts: one online survey conducted with a general population convenience sample and qualitative evaluation of translated items conducted within semi-structured interviews included in a larger interview study with participants from purposively sampled social groups.

### Survey study

#### Participants.

Recruitment took place from February to August of 2023 through social media platforms as well as physical advertisements within larger cities in Southern Sweden. Inclusion criteria consisted of being over 18 years of age and being able to respond to the survey in Swedish. In total, 509 participants were included in the sample. No careless responders were identified using indicators suggested by Ward and Meade [34]. Participants with more than 25% missingness for the NSSS-S items were excluded, leaving a final sample of 475 participants. There was a total of 43 items missing for 35 participants with each between 1–2 missing items. A Little’s MCAR test indicated that the missing data occurred completely at random, χ^2^(201) = 151.78, **p* *= .99, and missing values were therefore imputed using an expectation-maximization likelihood algorithm. Participant gender and sexual orientation were reported through free-text measures [35]. The sample included mainly women (71%) and individuals currently in a romantic relationship (74%), while there was greater diversity in terms of sexual orientation with around half the sample reporting a non-heterosexual sexual orientation. See Table 1 for complete sample demographics.

**Table 1:** Demographic information for survey sample participants (N = 475).

**Gender**	
Women	71% (339)
Men	20% (98)
Nonbinary/queer/genderfluid/demi	8% (39)
Intersex	0.2% (1)
Agender	0.6% (3)
Uncategorised	0.4% (2)
**Age**	
Min–Max	19–77
*M* (*SD*)	39.60 (11.00)
**Trans experience**	
Yes	10% (45)
No	89% (423)
Missing information	2% (7)
**Relationship status**	
Single	26% (124)
Partnered	74% (349)
Missing	0.4% (2)
**Relationship length (if partnered)**	
<1 year	26
Min–Max (for > 1 year)	1–55 years
*M*_ > 1 year_ (*SD*_ > 1 year_)	10.30 (9.42)
**Sexual orientation**	
Heterosexual	52% (247)
Lesbian/gay/homosexual	6% (26)
Bisexual/pansexual/multisexual	38% (179)
Asexual	0.8% (4)
Queer	3% (14)
Demisexual	0.2% (1)
Uncategorised answers	1% (5)
**Occupation**	
Employed	69% (328)
Student	16% (74)
Retired	3% (12)
Unemployed	4% (18)
Sick leave	3% (16)
Home with children	3% (13)
Self-employed	2% (7)
Other	1% (5)
**Education level**	
Doctoral degree	6% (29)
Licentiate degree	0.2% (1)
Master’s degree	40% (189)
Bachelor’s degree	34% (162)
High school degree	9% (43)
Elementary school education	0.2% (1)
Professional degree	6% (28)
Other adult education	5% (22)

Participants can be included in multiple categories of gender and sexual orientation. In total 12 participants reported a gender identity belonging to more than one category, and three participants reported a sexual orientation belonging to more than one category. Gender and sexual orientation group labels are based on participant self-identification.

#### Procedure.

The survey was hosted on Qualtrics (qualtrics.com) and included demographic questions (age, gender, sexual orientation, trans experience, relationship status, relationship length, education level, and occupation), a single-item measure of sexual satisfaction, and the Swedish version of the NSSS-S. Participants were informed about the purpose of the study, that no identifying information would be collected (e.g., IP address), and that they could choose to skip any questions that they did not wish to answer before providing informed consent to participate. Because the survey link was distributed in a way that ensured complete anonymity due to the sensitive topic of study, there were no formal ways of prohibiting multiple responses by the same person. However, as participants did not receive any compensation for participation the motivation to provide multiple responses was low. The survey was part of a larger research project and as such included additional measures not relevant for the current study. The median time to complete the full survey was 31 minutes.

#### Instruments.

**Swedish NSSS-S.** The NSSS-S [25] consists of 12 items which respondents evaluate on a Likert-scale ranging from 1 (*Not at all satisfied*) to 5 (*Extremely satisfied)* for their sex life during the last six months. The scale is scored using item sums, resulting in a possible range of scores from 12–60 with higher number indicating higher sexual satisfaction. The scale can further be divided into one Ego-Centred (items 1–6) and one Partner- and Activity-Centred subscale (items 7–12). Since the NSSS-S includes items related to satisfaction with partner-related aspects, the following instructions (in Swedish) where added as clarification for those who currently have several or no sexual partners: “If you have/have had several partners during the last six months, pick one of these and answer based on that one. If you have not had a partner during the last six months, answer based on your last partner or in relation to people you are generally attracted to”.

Following three steps recommended for the translation of self-report measures [33], instructions, response options, and items for the English NSSS-S were first translated from English to Swedish by TL. TL is a clinical psychologist, have additional training in sexology and do research in Swedish as well as English and as such has suitable expertise for the translation task. The measure was then back-translated into English by AK, who is a Swedish-English bilingual researcher but whose primary expertise is not in the field of sexology and could therefore serve as a more naïve translator [33]. The two translations where synthesized by TL and AK and in this process only minor inconsistencies were identified, which were due to occasional differences in verb-noun order between Swedish and English. For instance, item 4 in the original version reads as “The sexual function of my body” which was back-translated as “My body’s sexual functioning”. The synthesised translation was then evaluated by the entire research team in terms of semantic, idiomatic, experiential, and conceptual equivalence [33]. See supplementary File S1 for the Swedish NSSS-S and S2 for the English NSSS-S.

**Single-item measure of sexual satisfaction.** As there is no composite measure of sexual satisfaction available in Swedish to use as an indicator of convergent validity of the translated NSSS-S, a single-item measure of sexual satisfaction from the Life Satisfaction Scale [20] was included as it is the most evaluated measure of sexual satisfaction currently available in Swedish. The item read (in Swedish): “How satisfying would you say your sex life is on a scale from 1 (very dissatisfying) to 6 (very satisfying)?”.

#### Data analysis.

The factor structure of the NSSS-S Swedish version was compared to the two-factor structure established for the English and Croatian long version of NSSS [2], the NSSS-S in English [23], Spanish [26], and the NSSS-S in Portuguese [27]. Furthermore, we tested this structure against the unidimensional structure found for the English [25] and German [29] versions of NSSS-S. This was done through confirmatory factor analyses (CFAs) with robust maximum likelihood estimation using the package *lavaan* [36] in R version 4.3.2 [37]. Model fit was evaluated through the Comparative Fit Index (CFI), Root Mean Squared Error of Approximation (RMSEA), and the Standardised Root Mean Square Residual (SRMR). Good fit is defined as CFI > .95, RMSEA < .06, and SRMR < .05 [38] while acceptable fit is defined as CFI > .90, RMSEA < .08 [39], SRMR < .08 [38]. To test convergent evidence for validity, we also examined the correlation between the Swedish NSSS-S and a single-item measure of sexual satisfaction from the Life Satisfaction Scale [20].

### Interview study

#### Interview respondents.

In total, 20 respondents participated in the interviews and recruitment was performed on social media platforms and physical advertisements around Lund University. An invitation to participate was also distributed to university students through student administrators at different universities around Sweden. As interviews were conducted within a larger interview study on sexual satisfaction among social groups in Sweden, respondents represent two waves of purposeful sampling for individuals with experience of being prescribed anti-depressant medication and having experienced side effects that impacted their sexuality (**n* *= 11) and people above the age of 45 years currently going through or having gone through menopause (**n* *= 9). Interviews were conducted during September-November 2022 and 2023. These groups were chosen as they were likely to have experienced fluctuation in their sexual satisfaction due to physical effects of anti-depressant medication or menopause and therefore have valuable perspectives on how to measure different levels of sexual satisfaction. Respondents’ ages ranged from 20–62 years **(M* *= 40.50 years) and all respondents had some form of educational experience after high school, either at university or a professional degree. In total, 16 respondents were cisgendered women, three respondents were cisgendered men, and one respondent had a nonbinary gender identity. For sexual orientation, 15 respondents identified as heterosexual, three as bisexual, and two as lesbian women. All respondents except one were either in a romantic relationship or actively engaged in dating which involved partnered sex.

#### Procedure.

The interview respondents were instructed to respond freely to the NSSS-S items at the end of the semi-structured interview. The full interviews lasted on average 63.5 minutes. Respondents were asked the NSSS-S items in an open format, which included some rephrasing of questions to not rely on a bipolar answer scale but rather ask for how they evaluated the different aspects of sexual satisfaction. For example, the item “My partner’s sexual creativity” was rephrased to “How satisfied are you with your partner’s sexual creativity?”. Following presentation of all items, the face validity of the translated measure was assessed by respondents being asked to evaluate the extent to which they felt that the items captured important dimensions of sexual satisfaction.

#### Data analysis.

Respondent responses to NSSS-S items and their evaluation of the items as a measure of sexual satisfaction were analysed using summative content analysis which is based on interpretation of the latent content rather than only analysis of frequency of responses [40]. Responses were coded into two categories based on if respondents answered the question without issue or if they in some way problematized the question. Respondents were also asked if they found the scale suitable for measuring sexual satisfaction, including if any specific item felt unsuitable or if they had suggestions for aspects to add. These responses were initially coded into answers expressing that the respondent had nothing further to add and answers including further elaboration of views on the scale, and elaborated answers were further coded into content descriptive categories (e.g., “expresses difficulty understanding the wording of the question”).

### Ethics

The research was conducted in accordance with the Declaration of Helsinki [41] and was approved by the Swedish Ethical Review Authority (Dnr 2022-03724-01). See supplementary File S3 regarding compliance with PLOS one ethical guidelines for research with human participants.

## Results

### Survey study

Item descriptive statistics, item correlations, corrected item-total correlations, and item correlations to the single-item measure of sexual satisfaction are reported in Table 2. The summed full NSSS-S Swedish scale correlated strongly with the single-item measure of sexual satisfaction, **r* *= 0.74, **p* *< .001, as did the Ego-Centred, **r* *= 0.60, **p* *< .001, and Partner- and Activity-Centred, **r* *= 0.76, **p* *< .001, subscales. The full sample mean (*M* = 40.94, *SD* = 9.54) was significantly higher than the scale midpoint score of 36, *t*(474) = 11.30, **p* *< .001, *d = *0.52, indicating tha*t* the sample on a group level felt more sexually satisfied than unsatisfied. Furthermore, scores for the Ego-Centred subscale (*M* = 21.29, *SD* = 4.98) were significantly higher than for the Partner- and Activity-Centred subscale *(M* = 19.65, *SD *= 5.46)*, t*(474) = 8.39, **p* *< .001, **d* *= 0.39, indicating that the Swedish NSSS-S can differentiate between two related but separate aspects of sexual satisfaction.

**Table 2:** Descriptives and item correlations and corrected item-total correlations for NSSS-S items.

	*M (SD)*	NSSS-S items												
		CIT	1.	2.	3.	4.	5.	6.	7.	8.	9.	10.	11.	12.
1.	3.50 (1.17)	0.57	–											
2.	3.36 (1.21)	0.61	0.46	–										
3.	3.53 (1.14)	0.72	0.46	0.57	–									
4.	3.47 (1.09)	0.50	0.47	0.57	0.53	–								
5.	3.79 (1.01)	0.66	0.51	0.52	0.60	0.46	–							
6.	3.64 (0.99)	0.60	0.31	0.43	0.47	0.34	0.44	–						
7.	3.51 (1.17)	0.72	0.50	0.44	0.53	0.33	0.50	0.44	–					
8.	3.33 (1.19)	0.64	0.33	0.32	0.47	0.18	0.46	0.42	0.61	–				
9.	3.87 (1.06)	0.45	0.24	0.21	0.31	0.17	0.25	0.49	0.41	0.39	–			
10.	3.19 (1.20)	0.70	0.38	0.38	0.53	0.24	0.45	0.40	0.62	0.66	0.40	–		
11.	3.09 (1.15)	0.76	0.44	0.45	0.57	0.36	0.48	0.52	0.62	0.54	0.37	0.69	–	
12.	2.67 (1.24)	0.63	0.36	0.38	0.46	0.31	0.43	0.38	0.46	0.49	0.30	0.58	0.67	–
SSS	3.81 (1.44)	–	0.41	0.44	0.55	0.37	0.48	0.44	0.53	0.59	0.39	0.61	0.67	0.72

All correlations were significant at the **p* *< .001 level. CIT = corrected item-total correlations, SSS = sexual satisfaction, single-item.

Bartlett’s test of sphericity [42] and a Kaiser-Meyer-Olkin test [43] were used to test the factorability of the NSSS-S responses, as recommended by Dziuban and Shirkey [44]. Both tests indicated that the data was suitable for factor analysis: Bartlett’s test: χ^2^(78)= 3331.59, **p* *< .001. Kaiser-Meyer-Olkin test: MSA = 0.92. Scatter plots indicated that factor loadings showed a linear relationship to the latent variable(s) and that there was a linear relationship between Ego- and Partner- and Activity centred factors [45]. Mardia’s test for multivariate linearity [46] showed significant skewness and kurtosis among the NSSS-S items (**p* *< .001) and the assumption of multivariate normality among the indicators was therefore rejected. A robust maximum likelihood estimator was therefore used in the CFAs as it has been shown to handle issues of non-normality well [47].

The single-factor model showed generally poor fit, CFI = 0.85, RMSEA = 0.13 (90%CI [0.12, 0.14]), SRMR = 0.08, indicating that the Swedish NSSS-S does not have a unidimensional structure. Based on the existing split of items into Ego-centred and Partner- and Activity-centred subscales, a two-factor model with items 1–6 representing one factor and items 7–12 representing another factor was tested. This two-factor model displayed an acceptable CFI of 0.92 and SRMR of 0.06, but an absolute RMSEA value above the cut-off for acceptable fit, 0.10 (90%CI [0.08, 0.11]). The two-factor model was, however, a significantly better fit than the single-factor model, χ2(54) = 513.46, p < .001. The two factors showed a significant correlation with each other, *r* = 0.52, **z* *= 8.98, **p* *< .001. See Table 3 for standardised factor loadings of all items.

**Table 3:** Standardised factor loadings and R^2^ for two-factor models with uncorrelated and correlated item residuals.

		Two-factor CFA, uncorrelated item residuals			Two-factor CFA, correlated item residuals		
		Standardised factor loadings		*R* ^2^	Standardised factor loadings		*R* ^2^
English item	Swedish item	Ego-Centred subscale	Partner- and Activity-Centred subscale		Ego-Centred subscale	Partner- and Activity-Centred subscale	
1. The quality of my orgasms	Kvaliteten i mina orgasmer	0.63		0.40	0.62		0.39
2. My “letting go” and surrender to sexual pleasure during sex	Hur jag”släpper taget” och hänger mig till sexuell njutning under sex	0.72		0.51	0.69		0.48
3. The way I sexually react to my partner	På vilket sätt jag sexuellt reagerar på min partner	0.80		0.65	0.81		0.66
4. My body’s sexual functioning	Min kropps sexuella funktion	0.64		0.41	0.60		0.46
5. My mood after sexual activity	Min sinnesstämning efter sexuell aktivitet	0.75		0.56	0.75		0.57
6. The pleasure I provide to my partner	Njutningen som jag ger min partner	0.60		0.36	0.60		0.37
7. The balance between what I give and receive in sex	Balansen mellan vad jag ger och får vid sex		0.77	0.59		0.79	0.62
8. My partner’s emotional opening up during sex	Hur min partner känslomässigt öppnar upp under sex		0.73	0.53		0.72	0.52
9. My partner’s ability to orgasm	Min partners förmåga att få orgasm		0.48	0.24		0.47	0.22
10. My partner’s sexual creativity	Min partners sexuella kreativitet		0.82	0.67		0.81	0.65
11. The variety of my sexual activities	Variationen av mina sexuella aktiviteter		0.84	0.70		0.81	0.66
12. The frequency of my sexual activity	Frekvensen av min sexuella aktivitet		0.71	0.50		0.67	0.44

Since CFI and SRMR indicated that the model had acceptable fit while the RMSEA indicated mediocre fit, modification indices (MI) together with standardised expected parameter change values (SEPC) were inspected with the aim of identifying misspecifications of the model [48,49]. Because the factor structure was theoretically determined, only MIs regarding correlated residuals were considered and since all items where unidirectional only positive SEPC values where evaluated. Table 4 shows MI and SEPC values for correlated item residuals when the MI was significant and the SEPC was positive and above the suggested cut-off of 0.2 [48].

**Table 4:** Modification indices and standardised parameter change values (SEPC) for parameters considered as potential model misspecifications.

Parameter	Modification index	SEPC
Item 6 ~~ Item 9	53.22	0.35
Item 11 ~~ Item 12	36.43	0.36
Item 2 ~~ Item 4	28.70	0.29
Item 8 ~~ Item 10	18.27	0.26
Item 1 ~~ Item 7	14.86	0.20

The modified two-factor model allowing correlated item residuals, as specified in Table 3, displayed a good CFI of.97 and SRMR of.04, and an acceptable RMSEA value, 0.07 (90%CI [0.05, 0.08]). The modified two-factor model was a significantly better fit than the original two-factor model, χ2(53) = 303.82, p < .001.

As an additional test of internal consistency McDonald’s ω was calculated using maximum likelihood estimation. The NSSS-S showed high total reliability, ω_t_ = .93, and 71% of the variance in scores was attributable to a general factor of sexual satisfaction, ω_h_ = .71. This indicates that approximately 22% of the variance in scores represents something not related to the latent general construct of sexual satisfaction [50]. The Ego-centred and Partner- and Activity-centred subscales similarly showed high total reliability, ω_t_ = .87 and ω_t_ = .90.

#### Measurement invariance.

The NSSS-S includes items related to a sexual partner, and its measurement invariance based on relationship status has not been evaluated. To test if NSSS-S scores for those in a sexual relationship and those not in a sexual relationship relate similarly to the latent construct of sexual satisfaction we conducted multi-group CFAs for participants currently in a relationship (**n* *= 349) and participants not currently in a relationship (**n* *= 124) following the steps recommended by van de Schoot et al. [51]. These steps establish if the same factor structure is present in the different groups (configural invariance), if respondents in different groups give the same meaning to the latent construct (metric invariance), if the meaning and levels of the construct has the same meaning across groups (scalar invariance), and if the latent construct is measured with the same meaning, levels, and error variance across groups (strict invariance).

A CFA with unconstrained factor loadings and intercepts, testing configural invariance, showed acceptable fit in terms of CFI and SRMS but failed to reach the cut-off for RMSEA (see Table 5 for fit indices for all invariance models). A CFA with constrained factor loadings and unconstrained intercepts, testing metric invariance, showed comparable fit indices and did not differ significantly from the unconstrained model, Δχ^2^(10) = 11.78, *p* = 0.30. A CFA with constrained factor loadings and intercepts, testing scalar invariance, showed significantly worse fit than the metric invariance model, Δχ^2^(10) = 43.40, *p* < .001, indicating a difference in intercepts across groups for certain items. Investigating parameters decreasing model fit showed that the releasing the intercepts for items 1, 4, 5, and 11 would increase model fit. Item intercepts were released one by one in order of degree of significance, and a CFA with unrestrained intercepts for items 1, 4, and 11 no longer differed in terms of fit from the metric invariance model, Δχ^2^(7) = 7.60, *p* = 0.37, and partial scalar invariance was therefore reached. Finally, a CFA with constrained factor loadings, intercepts (excepting the intercepts allowed to vary in the scalar model) and residuals, testing strict invariance, was created and did not differ significantly in terms of fit from the partial scalar invariance model, Δχ^2^(12) = 8.88, *p* = 0.71. Based on the model for strict invariance, mean factor scores were compared between groups. Those currently in a sexual relationship had higher mean factor scores for the Ego-centred factor, *M*_diff_ = 0.20, t(208.25) = 2.81, **p* *= .01, **d* *= 0.29, and the Partner/Activity-centred factor, *M*_diff _= 0.52, t(225.69) = 6.21, **p* *< .001, **d* *= 0.64.

**Table 5:** Fit indices for multi-group CFAs testing measurement invariance based on relationship status.

	CFI	SRMS	Robust RMSEA [95% CI]
Configural invariance	0.91	0.06	0.10 [0.09:0.11]
Metric invariance	0.91	0.07	0.10 [0.08:0.11]
Scalar invariance	0.90	0.07	0.10 [0.08:0.11]
Partial scalar invariance	0.91	0.07	0.09 [0.08:0.11]
Strict invariance	0.92	0.07	0.09 [0.08:0.11]

CFI = Comparative Fit Index, SRMS = Standardized Root Mean Square Residual, RMSEA = Root Mean Square Error of Approximation

### Interview study

Of the total number of responses to the NSSS-S items across all respondents, 78% included a direct answer to the question without issue with the remaining responses including some form of meta-communication about the question. The most common reasons were that respondents found the question hard to answer because it would depend on situational factors (12% of responses) or vary over time (2% of responses). Only 4% of cases were due to requests for minor clarification of the wording of the question, indicating that the translation was generally well understood by participants.

Respondents were also asked if they found the scale suitable for measuring sexual satisfaction, including if any specific item felt unsuitable or if they had suggestions for aspects to add. No item was evaluated as directly unsuitable to measure sexual satisfaction, but three respondents did express that the large focus of the scale on orgasms as well as partnered sex lead to limitations in its usefulness. For instance, “sexual satisfaction can be so much else other than just orgasms” and “Why does ‘partner’ have to be there all the time? I mean, can’t sexual pleasure also come from your own sexuality?”. For specifically item 9 (“Partner’s ability to orgasm”), most respondents answered with short statements of being satisfied or not satisfied with their partner’s ability to orgasm. However, those respondents who gave more elaborate answers spoke of their partner’s ability to orgasm being related to their own feelings of having brought sexual satisfaction to their partner, but not as necessarily related to their own feelings of satisfaction. That is, feeling that they contributed to partner satisfaction in terms of partner orgasm was not seen as directly related to personal satisfaction (“How satisfied am I with my partner’s orgasms, for instance. I’m like, what? […] It can give me satisfaction as well but is this really about me […]”). Instead, some respondents described partner’s ability to orgasm as something beyond their control and as more influenced by factors inherent to the partner, such as for example the presence of gender dysphoria (“She doesn’t orgasm, given that she’s trans. So I actually just mostly feel sorry for her if she orgasms. Because I know it’s something she thinks is pretty uncomfortable. So I long, in that way, I long for the surgery in that sense. Then I can feel pleased when she has orgasms. Because then I know she is completely satisfied.”).

Suggestions for items to add consisted of questions asking about how aspects of the general relationship to a sexual partner influences the experience and purpose of sexual interaction, the influences of sexual norms on satisfaction, relating aspects of sexual satisfaction to levels of personal lust, and the representation of situational and temporal variation. The general aspects of the relationship that were suggested to be added were questions regarding the degree of openness and confidence one feels in communicating about sex with one’s partner, the level of reciprocal desire and passion present in the relationship, and how sexual practices contribute to feelings of emotional intimacy beyond direct sexual satisfaction. Suggested questions to be added in relation to sexual norms included the extent to which one’s ideal image of sex matches one’s actual sexual experiences. Finally, respondents expressed that the item regarding frequency of sexual activity should be rephrased to specify if one is satisfied with the frequency of sexual activity *given current levels of lust* (“There are not that many specific questions about lust, for example. [...] in my case, I don’t need to have a whole lot of sex to feel satisfied.”) and that the items were not well positioned to represent how aspects of sexual satisfaction varies with specific sexual encounters and changes in desire over time.

Respondents also expressed that the act of answering the items from the NSSS-S was in itself a valuable experience, allowing them to reflect on aspects of their sexual satisfaction. Highlighting the insights that responding to the scale brought, one respondent stated that:”I become a bit sad because it does also become so very clear that I don’t actually have any active sex life with my partner, and that became very clear with your questions […]”.

## Discussion

The Swedish version of the NSSS-S displayed a similar bi-dimensional factor structure as previously found for the Spanish, Portuguese, and English NSSS-S [23,26,27]. While the two-factor structure did not display optimal values for all fit measures, model fit was between good and acceptable for all indicators and substantially better than the one-factor model reported for the English [25] and German [29] versions. These findings support that scores for the NSSS-S should primarily be calculated on the subscale level to reflect the bi-dimensional structure of the measure. However, the latent level correlation between the two factors was substantial, indicating that Ego-Centred and Partner- and Activity-Centred aspects of sexual satisfaction are not two independent aspects of sexual satisfaction but are either, to some extent, reliant on each other or are both dependent on external factor(s). This latent level correlation is lower than those found for the Spanish [26], French [28], and German [29] versions of the NSSS-S, as well as lower than manifest subscale correlations for the Portugese version [27]. However, it is comparable to the factor correlations found in Croatian and US samples during the development of the NSSS [2]. These differences in strength of subscale relation across language versions are important to bear in mind for future cross-cultural comparisons of levels of sexual satisfaction using the NSSS-S, as it may indicate a need for different interpretation of subscale scores across cultures. Internal reliability was high for the total measure with 71% of the variance accounted for by a general factor of sexual satisfaction. People currently in a relationship reported higher levels of sexual satisfaction than singles, supporting the ability of the Swedish NSSS-S to differentiate between groups previously identified as differing in sexual satisfaction [52–54]. The qualitative evaluation of the scale translation did not reveal any marked difficulty in understanding and responding to the Swedish version of the NSSS-S, indicating that the translation was of sufficient quality.

While the Swedish NSSS-S displayed a generally acceptable fit to a two-factor model, the fit was substantially improved by including correlated item residuals between five pairs of items. Since there are several reasons why item residuals may covary, including a shared method factor or a shared correlation with an omitted variable [55], conducting post hoc model modifications represents a departure from a confirmatory framework into an exploratory one and increases the risk of overfitting [56,57]. Potential reasons for why item errors might vary in a systematic manner should therefore be further explored in additional samples and relevant factors to explore include personal sexual function as well as that of latest or current sexual partner, relationship quality, cohabitation status with partner, and general life stressors [5]. Furthermore, the Swedish NSSS-S showed partial scalar invariance based on relationship status but achieving good fit for both groups required non-constrained intercepts for items 1, 4, and 11. This indicates that the meanings of those items might differ based on relationship status, which is an aspect of the NSSS-S that has not previously been evaluated. How unpartnered individuals define a satisfying sex life and what contributes to sexual satisfaction among unpartnered individuals is an under researched topic, as sexual satisfaction has mainly been studied within the context of romantic relationships [58]. In the current study, the Swedish NSSS-S displayed metric invariance, indicating that all items showed a similar relation to the latent variable of sexual satisfaction for both partnered and unpartnered individuals. This supports existing findings that aspects related to sexual satisfaction are similar across relationship status, given that contentment with level of sexual activity and contentment with current relationship/singlehood is accounted for [58]. However, the partial scalar invariance found in the current study indicates that partnered and unpartnered individuals with similar total scores on the NSSS-S show systematic differences in their levels of satisfaction with orgasm quality (higher among partnered individuals), their body’s sexual functioning (higher among unpartnered individuals), and the variety of their sexual activities (higher among unpartnered individuals). That is, partnered individuals reported greater satisfaction with the quality of their orgasms, while unpartnered individuals reported higher satisfaction with their body’s sexual functioning and the variety of their sexual activities, but these differences for specific items were not related to overall group differences in levels of sexual satisfaction. Relationship to sexual partners, types of sexual activities, and levels of solo sex have been shown to differ among partnered and unpartnered individuals [58–60], and further research is needed to establish how such differences may influence satisfaction with specific aspects of general sexual satisfaction. Despite only partial scalar invariance being reached, it is still possible to use the Swedish NSSS-S as a measure of sexual satisfaction for both partnered and non-partnered individuals, as current recommendations state that as long as at least two loadings and intercepts are constrained across groups it is still possible to make valid inferences about group differences in latent factor means [51].

As in validations of the NSSS-S in English and Spanish [23,26], item 9 (Partner’s ability to orgasm) loaded less strongly on the intended Partner- and Activity-Centred factor. This raises the question of how much perceptions of partner’s ability to orgasm contributes to personal sexual satisfaction, which was also highlighted in the qualitative responses to the item. This weaker loading could be due to beliefs regarding causes for partner’s ability to orgasm. That is, if one sees partner’s ability to orgasm as being caused by factors external to the self its connection to experiences of personal sexual satisfaction may diminish. This potential interpretation is supported by aspects brought up by respondents in the interview study regarding the separation between own satisfaction and contributions to partner satisfaction. Therefore, in agreement with Mark et al. [23], we recommend further examination of when and how perceptions of a sexual partner’s ability to orgasm relates to different aspects of sexual satisfaction. The relationship between partner orgasm and sexual satisfaction may be more complex and connected to interpersonal and gendered aspects than was accounted for in the development of the NSSS-S. Perceiving that your partner wants you to orgasm is related to higher levels of sexual satisfaction for both women and men [61], but actual partner orgasm consistency has no added benefit for sexual satisfaction beyond that of personal orgasm consistency [62]. Whether or not partner orgasm matters for personal sexual satisfaction may, however, be a gendered phenomenon. There is some indication that men who have sex with women report lower sexual satisfaction if their partner orgasms more consistently than they themselves do, while the high generally high level of orgasm among men may make this a less salient feature for their women partners [62]. Gendered social norms can further influence the extent to which someone evaluates their sexual satisfaction based on perceptions of partner satisfaction or based on their own direct physical experience of the sexual encounter, where heterosexual women show a greater tendency to base their sense of satisfaction on whether or not their male partner was satisfied [63]. A primary concern with partner satisfaction has also been reported by men who have sex with men [63], while bisexual women report expecting to orgasm to a higher degree when having sex with a woman compared to with a man [64]. In contrast, men having sex with women pursue the goal to make their partner orgasm to a lower degree than women, despite a stronger feeling that their partner wants them to orgasm [61]. Experimental research on imagined sexual situations indicate that heterosexual men feel more masculine when imagining that a woman sexual partner orgasmed, and that this was particularly pronounced for men that were insecure about if they fulfilled a masculine gender role [65]. Taken together, this research indicates that partner orgasm may be of importance for personal sexual satisfaction, but that this would depend on aspects such as partner gender, expectation of orgasm, perceived partner pursuit of orgasm, and centrality of a masculine gender identity. Given these complex dynamics of gendered expectations, a fruitful avenue of future research would be to examine the ways that different social groups use discourses of gender to position their sexual satisfaction, including the relation between sexual non-normativity and experiences of sexual stigma [22].

The translated version of the NSSS-S showed convergent validity through its high correlation with the single-item measure of sexual satisfaction. Despite the similar results for the single-item measure and the NSSS-S in terms of overall sexual satisfaction, the bi-dimensional structure of the NSSS-S was able to show a sample difference between the Ego-Centred and Partner- and Activity-Centred subscales, as well as group differences between partnered and non-partnered individuals for either subscale. This indicates that using the NSSS-S can provide more nuanced information regarding different aspects of sexual satisfaction than a single-item measure can. This increase in ability to discern different contributors to sexual satisfaction justifies the added participant effort in responding to a full scale compared to a single-item, especially given that the NSSS-S is in itself a short scale.

While sexual satisfaction is often defined as the degree of positive affect associated with sex, other definitions state that sexual satisfaction is not an absolute evaluation, but rather “the degree to which a person’s sexual activity meets his or her expectations” ([66], p. 62). The NSSS-S seeks to measure sexual satisfaction as the absolute affective evaluation of three dimensions of sexuality: individual, interpersonal, and behavioural aspects [2]. That is, as the degree to which one is pleased or not pleased with specific aspects of sexual experience. However, qualitative evaluation of the NSSS-S in this study brought up concerns that the absolute approach does not adequately represent the relative nature of evaluations of satisfaction, including situational and temporal factors. Some respondents spoke instead of evaluation of sexual satisfaction as something that takes place in relation to current needs and social norms regarding what a satisfying sex life is; expressing the view that sexual satisfaction is related to expectations rather than being absolute. This perspective aligns with a definition of sexual satisfaction that includes both the state-like momentary aspect of positive affect during sexual activity and a trait-like general tendency to enjoy sexual activities [67]. Trait and state aspects of sexual satisfaction are suggested to interact bidirectionally and in this way influence which rewards are expected from a sexual encounter and the evaluation of if these expectations were fulfilled [67]. Expectations regarding what is or is not satisfying sexual activity can be highly influenced by social norms, for instance through creating an imperative to engage in vaginal penetrative intercourse [68] or that partner satisfaction should be prioritised at the expense of personal satisfaction [22,63]. In addition, some participants also suggested in their qualitative responses how sexual satisfaction can be related to more general factors of the relationship, such as reciprocity, the quality of communication and emotional intimacy. The intricate relationship between sexual satisfaction and more general aspects of the relationship, such as relationship satisfaction, has been highlighted in research as well [69].

Future research should therefore further examine how evaluation of sexual satisfaction using standardized measures relates to perceptions of social norms and relationship quality regarding sexual activity. Additionally, further longitudinal research on how trait and state aspects of sexual satisfaction relate to each other can help inform how situational, temporal, and relational variability relates to general satisfaction levels. It is possible that the NSSS-S can be used within such research as a complement to measurement of additional concepts related to expected satisfaction.

Frequency and variety of sexual acts have previously been identified as an important predictor for sexual satisfaction [5] and there was a strong connection between latent sexual satisfaction and NSSS-S items asking about satisfaction with variety and frequency of sexual acts, which supports previous findings that higher frequency of sex and variety of sexual acts predict higher sexual satisfaction [5]. However, a criticism of the scale items from interview respondents was that frequency and variety of sex was not explicitly asked in relation to their current levels of sexual lust. Besides personal levels of lust, the frequency of sex seen as satisfactory is dependent on a dyadic process which balances own desired frequency with the frequency desired by a sexual partner [32]. It is possible that most participants in the survey study performed an evaluation of their satisfaction with frequency and variety of sexual activity in relation to their current levels of lust and partner’s desired frequency of sex without prompting, but the lack of explicit instruction to do so may increase the error variance in scale responses as it increases the risk of differential interpretation of the item [63]. This interpretation is further supported by the correlation of item residuals for these two items and could indicate that the NSSS-S partly underrepresents the construct of sexual satisfaction levels [70]. However, this discrepancy between the survey study and the interview study may also be due to sample differences since the survey sample was a general convenience sample while the interview sample consisted of respondents likely to experience some degree of clinically relevant sexual dysfunction due to side-effects of anti-depressant medication or physical aspects of menopause. It is possible that frequency of sexual activity has a stronger relationship to general sexual satisfaction for non-clinical populations, while factors inhibiting desire among clinical samples can decrease the importance of sexual frequency in favour of other aspects of sexual satisfaction.

There is evidence for the validity of the NSSS-S as a measure of sexual satisfaction in several languages [2,23,25–29], with the current study providing initial support for its use also in Swedish. The NSSS-S was strongly correlated to the single-item measure that is currently most commonly used in a Swedish context [15,16] but has the added benefit of allowing for more nuanced exploration of Ego-Centred and Partner- and Activity-Centred aspects of sexual satisfaction. However, as identified by the respondents in the interview study, the NSSS-S does share some of the issues common to all self-report measures seeking to measure a general level of a situationally and temporally variant concept such as sexual satisfaction [63]. Measuring sexual satisfaction over such a relatively large time period as the last six months risks losing the peaks and valleys of satisfaction with one’s sex life which can provide important information, especially if sexual satisfaction is being measured for therapeutic or intervention purposes [63]. Despite this issue, the NSSS-S was perceived as a scale that could have benefited respondents in identifying areas of their sex life that could be improved, including their relationship to their own sexuality and their relationship to their partner’s sexuality. This supports that the NSSS-S can be used for the health promoting purposes intended in its construction [2] and that have been shown in recent research using it to evaluate effects of couple’s therapy [71]. Furthermore, given its short format, the NSSS-S could be fruitfully included in future research using ecological momentary assessment [72]. Instructions to respond to the NSSS-S in relation to a shorter time span and repeated measurement points would potentially make the measure better suited to capture the temporal and situational variation in sexual satisfaction that was highlighted in the qualitative analysis.

### Limitations

A limitation of the current study is that both the survey study and interview study sample consisted mainly of heterosexual, university-educated women. This restricts our possibility to assess the suitability of the Swedish NSSS-S to measure sexual satisfaction among different social groups, particularly for sexual minorities and/or less highly educated population groups who can be less familiar with responding to self-report measures. Including interview respondents with a more diverse educational and cultural background would have provided stronger evidence that the translation of NSSS-S items resulted in an intelligible measure for different population groups. Gathering additional evidence for the validity of the NSSS-S as a measure of sexual satisfaction with these target groups would give much needed information about its suitability for the general population. Despite the skewness of the survey study sample, it did include a substantial proportion of bisexual people (38%) which increases the likelihood that the NSSS-S is a psychometrically sound measure also in this population. Additionally, the survey sample size was far larger than the minimum recommended number of participants for confirmatory factor analyses [73] and included valuable representation of participants from a wide age range.

Even with a sufficiently large number of participants, the generalizability of our findings is limited by the use of a convenience sample in the survey study. Convenience sampling carries the risk of influencing variable range through self-selection biases, where those with particularly high or particularly low sexual satisfaction may be especially interested in participating in a study on the topic. However, the current sample displayed no such bimodality in NSSS-S scores, and snowball samples recruited through social media have been shown to give similar psychometric results as other commonly used sampling methods (e.g., online panel samples or student samples; [74]). The use of a convenience sample further limits the generalizability of the findings of the current study as likelihood of participation was confounded with aspects such as education level and internet access [75]. Additional sampling limitations include that there was no technical safeguard against participants completing the survey multiple times, and that no measure of nationality was included. Ensuring generalizability of the findings that the Swedish NSSS-S is a psychometrically sound and semantically valid measure of sexual satisfaction among a Swedish speaking population therefore requires additional testing in random, or carefully stratified, samples.

Previous research on sexual satisfaction has shown that personal definitions of sexual satisfaction can differ based on aspect such as gender [22] and sexual orientation [76]. However, we were not able to test the measurement invariance of the Swedish version of the NSSS-S across gender and sexuality groups due to the small number of men in the sample and the heterogenous nature of sexual minority groups represented, resulting in group sizes too low for well powered analyses [77]. The NSSS was developed to be a suitable measure of sexual satisfaction regardless of respondent gender, and there is support that it functions similarly for women and men [2]. However, the NSSS-S has not been systematically examined in relation to gender and to ensure that NSSS-S items are interpreted similarly for all respondents, additional research is required which purposefully samples relevant social groups and conducts additional tests of measurement invariance.

To reach acceptable model fit as assessed by the RMSEA, it was necessary to allow free parameters to the CFA. Using modification indices to release parameters in this way decreases the reliability of all model parameters and is a practice facing increasing criticism [56]. The use of modification indices for improvement of model fit in the current study may be one reason for the lower subscale correlation found for the Swedish NSSS-S compared to other language versions of the measurement, as allowing correlated item residuals will additionally decrease the reliability of factor correlation estimates. The RMSEA is particularly sensitive to the number of degrees of freedom in a model as well as the number of participants and can underestimate fit in smaller models while overestimating it in larger models [78]. As the two-factor model with constrained parameters reached acceptable fit for the CFI and the SRMR but not for the RMSEA, further testing of the Swedish NSSS-S using additional indications of fit [79] is needed to assess potential causes of model misspecification and supply reliable model parameters, including examining the latent correlation between Partner/Activity-centred and Ego-centred sexual satisfaction in Swedish samples. This could also allow for an evaluation of the test-retest reliability of the Swedish NSSS-S, which was not assessed in the current study due to the use of a cross-sectional design. Until the measurement invariance of the Swedish NSSS-S by gender and sexual orientation has been tested and further validation has been conducted using a population representative sample, researchers should be cautious when interpreting the scale scores of the Swedish NSSS-S in samples that substantially differ from that of the current study. Conducting a meta-analysis would provide valuable information on how the NSSS-S generally performs with diverse population groups and allow for comparisons of psychometric properties across languages and cultures.

Finally, we used a single-item measure from the Life Satisfaction Scale [20] to assess evidence for convergence of scores of the translated NSSS-S with a previously used measure. Given the limitations of single-item measurements [22,23] it would have instead been preferable to compare NSSS-S scores to another multi-faceted composite measure such as the GMSEX [3]. However, as there are currently no other validated scale measures of sexual satisfaction in Swedish this was not an available option. Future research should therefore continue to develop measurements of sexual satisfaction in Swedish which can be compared and assessed in relation to each other, including translating and validating the GMSEX.

## Conclusions

The current study translated and evaluated a Swedish version of the NSSS-S and found that the measure shows good or acceptable psychometric properties, including the expected bi-dimensional factor structure, partial measurement invariance for relationship status, a strong association with a well-used single-item measure of sexual satisfaction, and the ability to differentiate between groups generally found to differ in sexual satisfaction levels. Qualitative evaluation of the translated items indicated no issues with understanding the wording of the measure. However, respondents expressed critical views stating that general measures of sexual satisfaction such as the NSSS-S does not necessarily capture the ways that satisfaction is situationally and temporally variant, showing the need for further measurement refinement in the field. These findings therefore support that the Swedish NSSS-S can be used to measure Ego-Centred and Partner- and Activity-Centred aspects of sexual satisfaction in a Swedish context.

## Supporting information

S1 File Swedish new sexual satisfaction scale–short form. (PDF)

S2 File New sexual satisfaction scale–short form. (PDF)

S3 File Human participants research checklist. (PDF)

## References

[1] World Health Organisation. Sexual health. 2022. https://www.who.int/health-topics/sexual-health#tab=tab_1

[2] Stulhofer A, Busko V, Brouillard P. Development and bicultural validation of the new sexual satisfaction scale. J Sex Res. 2010;47(4):257–68. doi: 10.1080/00224490903100561 19629836

[3] Lawrance K, Byers ES. Sexual satisfaction in long‐term heterosexual relationships: The interpersonal exchange model of sexual satisfaction. Personal Relationships. 1995;2(4):267–85. doi: 10.1111/j.1475-6811.1995.tb00092.x

[4] Shahhosseini Z, Gardeshi ZH, Pourasghar M, Salehi F. A review of affecting factors on sexual satisfaction in women. Mater Sociomed. 2014;26(6):378–81. doi: 10.5455/msm.2014.26.378-381 25685081 PMC4314168

[5] Sánchez-Fuentes M del M, Santos-Iglesias P, Sierra JC. A systematic review of sexual satisfaction. International Journal of Clinical and Health Psychology. 2014;14(1):67–75. doi: 10.1016/s1697-2600(14)70038-9

[6] Schmiedeberg C, Huyer-May B, Castiglioni L, Johnson MD. The More or the Better? How Sex Contributes to Life Satisfaction. Arch Sex Behav. 2017;46(2):465–73. doi: 10.1007/s10508-016-0843-y 27757732

[7] Flynn KE, Lin L, Bruner DW, Cyranowski JM, Hahn EA, Jeffery DD, et al. Sexual satisfaction and the importance of sexual health to quality of life throughout the life course of U.S. adults. J Sex Med. 2016;13(11):1642–50. doi: 10.1007/s10508-016-0843-y 27671968 PMC5075511

[8] Mitchell KR, Lewis R, O’Sullivan LF, Fortenberry JD. What is sexual wellbeing and why does it matter for public health?. Lancet Public Health. 2021;6(8):e608–13. doi: 10.1016/S2468-2667(21)00099-2 34166629 PMC7616985

[9] Seiter NS, Quirk K, Hardy N, Zinbarg RE, Goldsmith JZ, Pinsof WM. Changes in Commitment and Sexual Satisfaction: Trajectories in Couple Therapy. J Sex Marital Ther. 2020;46(3):296–302. doi: 10.1080/0092623X.2019.1677274 31661426

[10] The Public Health Authority. Sexuell och reproduktiv hälsa och rättigheter (SRHR) i Sverige 2017 - Resultat från befolkningsundersökningen SRHR2017. 2019. https://www.folkhalsomyndigheten.se/publikationer-och-material/publikationsarkiv/s/sexuell-och-reproduktiv-halsa-och-rattigheter-i-sverige-2017/

[11] del Mar Sánchez-Fuentes M, Sierra JC. Sexual satisfaction in a heterosexual and homosexual Spanish sample: the role of socio-demographic characteristics, health indicators, and relational factors. Sexual and Relationship Therapy. 2015;30(2):226–42. doi: 10.1080/14681994.2014.978275

[12] May A, Johnston KL. Ego-Centred and Partner/Activity-Focused Sexual Satisfaction: The Role of Self-Esteem and Sexual Assertiveness in Cisgender Heterosexual Women. Sex Roles. 2022;86(3–4):179–88. doi: 10.1007/s11199-021-01258-x

[13] The Public Health Authority. Nationell strategi för sexuell och reproduktiv hälsa och rättigheter: En god, jämlik och jämställd sexuell och reproduktiv hälsa i hela befolkningen. 2020. https://www.folkhalsomyndigheten.se/publikationer-och-material/publikationsarkiv/n/nationell-strategi-for-sexuell-och-reproduktiv-halsa-och-rattigheter-srhr/

[14] Beckman N, Waern M, Gustafson D, Skoog I. Secular trends in self reported sexual activity and satisfaction in Swedish 70 year olds: cross sectional survey of four populations, 1971-2001. BMJ. 2008;337(7662):a279. doi: 10.1136/bmj.a279 18614505 PMC2483873

[15] Schönnesson LN, Zeluf G, Garcia-Huidobro D, Ross MW, Eriksson LE, Ekström AM. Sexual (Dis)satisfaction and Its Contributors Among People Living with HIV Infection in Sweden. Arch Sex Behav. 2018;47(7):2007–26. doi: 10.1007/s10508-017-1106-2 29441436 PMC6097728

[16] Carlsson-Lalloo E, Rusner M, Berg M, Svedhem V, Mellgren Å. People living with HIV in Sweden report high levels of sexual satisfaction in a registry-based cohort study. AIDS Care. 2021;34(5):559–67. doi: 10.1080/09540121.2021.1909698 33793361

[17] Carlsson-Lalloo E, Berg M, Rusner M, Svedhem V, Mellgren Å. Sexual satisfaction and its associations with patient-reported outcomes in a cohort of women living with human immunodeficiency virus in Sweden. Int J STD AIDS. 2022;33(8):751–60. doi: 10.1177/09564624221100056 35622448

[18] Ek A-S, Holmström C, Elmerstig E. Sexuality >1 year after brain injury rehabilitation: A cross-sectional study in Sweden. Brain Inj. 2023;37(1):34–46. doi: 10.1080/02699052.2022.2145358 36408962

[19] Ostman M. Low satisfaction with sex life among people with severe mental illness living in a community. Psychiatry Res. 2014;216(3):340–5. doi: 10.1016/j.psychres.2014.02.009 24656521

[20] Fugl-Meyer AR, Bränholm I-B, Fugl-Meyer KS. Happiness and domain-specific life satisfaction in adult northern Swedes. Clin Rehabil. 1991;5(1):25–33. doi: 10.1177/026921559100500105

[21] Moncada I, Micheltorena CF, Martínez-Sánchez EM, Gutiérrez JR. Evaluation of the psychometric properties of the life satisfaction checklist as a screening tool for erectile dysfunction. J Sex Med. 2008;5(1):83–91. doi: 10.1111/j.1743-6109.2007.00640.x 18042219

[22] McClelland SI. Intimate Justice: A Critical Analysis of Sexual Satisfaction. Social and Personality Psychology Compass. 2010;4(9):663–80. doi: 10.1111/j.1751-9004.2010.00293.x

[23] Mark KP, Herbenick D, Fortenberry JD, Sanders S, Reece M. A psychometric comparison of three scales and a single-item measure to assess sexual satisfaction. J Sex Res. 2014;51(2):159–69. doi: 10.1080/00224499.2013.816261 24112135

[24] Hudson WW, Harrison DF, Crosscup PC. A short‐form scale to measure sexual discord in dyadic relationships. The Journal of Sex Research. 1981;17(2):157–74. doi: 10.1080/00224498109551110

[25] Brouillard P, Štulhofer A, Buško V. The new sexual satisfaction scale (NSSS) and its short form (NSSS-S). Handbook of sexuality-related measures. Taylor and Francis. 2019. 496–9.

[26] Strizzi J, Fernández-Agis I, Alarcón-Rodríguez R, Parrón-Carreño T. New Sexual Satisfaction Scale-Short Form--Spanish Version. PsycTESTS Dataset. American Psychological Association (APA). 2016. doi: 10.1037/t57449-000 26643768

[27] Santos Pechorro P, Monteiro Pascoal P, Neves Jesus S, Almeida AI, Soares Figueiredo C, Vieira RX. Propriedades psicométricas da versão portuguesa da Nova Escala de Satisfação Sexual – versão curta. Revista Internacional de Andrología. 2016;14(3):94–100. doi: 10.1016/j.androl.2016.04.006

[28] Gouvernet B. French Validation of the New Sexual Satisfaction Scale Short Form (NSSS-SF Fr). Sexes. 2024;5(1):31–45. doi: 10.3390/sexes5010003

[29] Hoy M, Strauß B, Kröger C, Brenk-Franz K. Evaluation of the German Short Version of the “New Sexual Satisfaction Scale” (NSSS-SD) in a Representative Sample. Psychother Psychosom Med Psychol. 2019;69(3–04):129–35. doi: 10.1055/a-0620-0002 29933463

[30] Arpacioglu S, Arpacioglu ZB, Avsar G. Greater mindfulness does not influence sexual satisfaction but is independently associated with lower relationship satisfaction. Sexual and Relationship Therapy. 2023;39(4):1254–72. doi: 10.1080/14681994.2023.2259821

[31] Palha-Fernandes E, Alves P, Lourenço M. Sexual satisfaction determinants and its relation with perfectionism: A cross-sectional study in an academic community. Sexual and Relationship Therapy. 2022;37(1):100–14. doi: 10.1080/14681994.2019.1677884

[32] Pascoal PM, Narciso I de SB, Pereira NM. What is sexual satisfaction? Thematic analysis of lay people’s definitions. J Sex Res. 2014;51(1):22–30. doi: 10.1080/00224499.2013.815149 24070214

[33] Beaton DE, Bombardier C, Guillemin F, Ferraz MB. Guidelines for the process of cross-cultural adaptation of self-report measures. Spine (Phila Pa 1976). 2000;25(24):3186–91. doi: 10.1097/00007632-200012150-00014 11124735

[34] Ward MK, Meade AW. Dealing with Careless Responding in Survey Data: Prevention, Identification, and Recommended Best Practices. Annu Rev Psychol. 2024;74:577–96. doi: 10.1146/annurev-psych-040422-045007 35973734

[35] Lindqvist A, Sendén MG, Renström EA. What is gender, anyway: a review of the options for operationalising gender. Psychology & Sexuality. 2020;12(4):332–44. doi: 10.1080/19419899.2020.1729844

[36] Rosseel Y. lavaan: AnRPackage for Structural Equation Modeling. J Stat Soft. 2012;48(2). doi: 10.18637/jss.v048.i02

[37] R Core T. R: A language and environment for statistical computing. 2023. https://www.r-project.org/

[38] Hu L, Bentler PM. Cutoff criteria for fit indexes in covariance structure analysis: Conventional criteria versus new alternatives. Structural Equation Modeling: A Multidisciplinary Journal. 1999;6(1):1–55. doi: 10.1080/10705519909540118

[39] Little TD. Longitudinal Structural Equation Modeling. New York: The Guildford Press; 2013.

[40] Hsieh H-F, Shannon SE. Three approaches to qualitative content analysis. Qual Health Res. 2005;15(9):1277–88. doi: 10.1177/1049732305276687 16204405

[41] World Medical Association. Declaration of Helsinki - Ethical principles for medical research involving human subjects. 2013. https://www.wma.net/policies-post/wma-declaration-of-helsinki-ethical-principles-for-medical-research-involving-human-subjects/10.1001/jama.2013.281053 24141714

[42] Bartlett MS. Tests of significance in factor analysis. British Journal of Statistical Psychology. 1950;3(2):77–85. doi: 10.1111/j.2044-8317.1950.tb00285.x

[43] Kaiser HF, Rice J. Little Jiffy, Mark Iv. Educational and Psychological Measurement. 1974;34(1):111–7. doi: 10.1177/001316447403400115

[44] Dziuban CD, Shirkey EC. When is a correlation matrix appropriate for factor analysis? Some decision rules. Psychological Bulletin. 1974;81(6):358–61. doi: 10.1037/h0036316

[45] Flora DB, Labrish C, Chalmers RP. Old and new ideas for data screening and assumption testing for exploratory and confirmatory factor analysis. Front Psychol. 2012;3:55. doi: 10.3389/fpsyg.2012.00055 22403561 PMC3290828

[46] Mardia KV. Measures of multivariate skewness and kurtosis with applications. Biometrika. 1970;57(3):519–30. doi: 10.1093/biomet/57.3.519

[47] Brown TA. Confirmatory factor analysis for applied research. New York: Guildford Publications. 2015.

[48] Whittaker TA. Using the modification index and standardized expected parameter change for model modification. J Exp Educ. 2012;80(1):26–44. doi: 10.2307/26594341

[49] Saris WE, Satorra A, van der Veld WM. Testing Structural Equation Models or Detection of Misspecifications?. Structural Equation Modeling: A Multidisciplinary Journal. 2009;16(4):561–82. doi: 10.1080/10705510903203433

[50] Revelle W, Condon DM. Reliability from α to ω: A tutorial. Psychol Assess. 2019;31(12):1395–411. doi: 10.1037/pas0000754 31380696

[51] van de Schoot R, Lugtig P, Hox J. A checklist for testing measurement invariance. European Journal of Developmental Psychology. 2012;9(4):486–92. doi: 10.1080/17405629.2012.686740

[52] Antičević V, Jokić‐Begić N, Britvić D. Sexual self‐concept, sexual satisfaction, and attachment among single and coupled individuals. Personal Relationships. 2017;24(4):858–68. doi: 10.1111/pere.12217

[53] Buczak-Stec E, König H-H, Hajek A. Sexual satisfaction of middle-aged and older adults: longitudinal findings from a nationally representative sample. Age Ageing. 2021;50(2):559–64. doi: 10.1093/ageing/afaa161 32960208

[54] Cranney S. Sex Life Satisfaction in Sub-Saharan Africa: A Descriptive and Exploratory Analysis. Arch Sex Behav. 2017;46(7):1961–72. doi: 10.1007/s10508-017-0984-7 28484861

[55] Gerbing DW, Anderson JC. On the Meaning of within-Factor Correlated Measurement Errors. J CONSUM RES. 1984;11(1):572. doi: 10.1086/208993

[56] Landis R, Edwards BD, Cortina J. On the practice of allowing correlated residuals among indicators in structural equation models. In: Lance CE, Vandenberg RJ. Statistical and Methodological Myths and Urban Legends Doctrine, Verity and Fable in Organizational and Social Sciences. New York: Routledge. 2009. 193–215.

[57] Schreiber JB, Nora A, Stage FK, Barlow EA, King J. Reporting Structural Equation Modeling and Confirmatory Factor Analysis Results: A Review. The Journal of Educational Research. 2006;99(6):323–38. doi: 10.3200/joer.99.6.323-338

[58] Fischer N. Singles Not Sexually Satisfied? Prevalence and Predictors of Sexual Satisfaction in Single versus Partnered Adults. Int J Sex Health. 2023;35(4):515–28. doi: 10.1080/19317611.2023.2241849 38596451 PMC10903649

[59] Furman W, Shaffer L. Romantic partners, friends, friends with benefits, and casual acquaintances as sexual partners. J Sex Res. 2011;48(6):554–64. doi: 10.1080/00224499.2010.535623 21128155 PMC3163778

[60] Kislev E. The Sexual Activity and Sexual Satisfaction of Singles in the Second Demographic Transition. Sex Res Soc Policy. 2021;18(3):726–38. doi: 10.1007/s13178-020-00496-0

[61] Wolfer C, Carmichael CL. Personal and perceived partner orgasm pursuit: A daily diary study about the gendered orgasm gap. Journal of Social and Personal Relationships. 2025;42(6):1265–90. doi: 10.1177/02654075251316579

[62] Leonhardt ND, Busby DM, Disalvo K, Hanna-Walker VR, Kim JJ, Willoughby BJ, et al. Orgasm Consistency in Mixed-Gender Couples: Actor, Partner, and Discrepancy Effects from Dyadic Response Surface Analysis. J Sex Res. 2024;61(2):216–27. doi: 10.1080/00224499.2023.2164883 36652377

[63] McClelland SI. Who is the “Self” in Self Reports of Sexual Satisfaction? Research and Policy Implications. Sex Res Soc Policy. 2011;8(4):304–20. doi: 10.1007/s13178-011-0067-9

[64] Dickman K, Wetzel GM, Sanchez DT. The Role of Partner Gender: How Sexual Expectations Shape the Pursuit of an Orgasm Goal for Heterosexual, Lesbian, and Bisexual Women. Social Psychological and Personality Science. 2024;16(5):495–506. doi: 10.1177/19485506241235235

[65] Chadwick SB, van Anders SM. Do Women’s Orgasms Function as a Masculinity Achievement for Men?. J Sex Res. 2017;54(9):1141–52. doi: 10.1080/00224499.2017.1283484 28276934

[66] DeLamater J. Emotions and sexuality. In: McKinney K, Sprecher S. Sexuality in close relationships. Mahwah: Lawrence Erlbaum Associates, Inc. 1991. 49–70.

[67] Werner M, Borgmann M, Laan E. Sexual Pleasure Matters - and How to Define and Assess It Too. A Conceptual Framework of Sexual Pleasure and the Sexual Response. Int J Sex Health. 2023;35(3):313–40. doi: 10.1080/19317611.2023.2212663 38595929 PMC10903685

[68] Ekholm E, Lundberg T, Carlsson J, Norberg J, Linton SJ, Flink IK. ”A lot to fall back on”: experiences of dyspareunia among queer women. Psychology & Sexuality. 2022;13(5):1242–55. doi: 10.1080/19419899.2021.2007988

[69] McNulty JK, Wenner CA, Fisher TD. Longitudinal Associations Among Relationship Satisfaction, Sexual Satisfaction, and Frequency of Sex in Early Marriage. Arch Sex Behav. 2016;45(1):85–97. doi: 10.1007/s10508-014-0444-6 25518817 PMC4472635

[70] American Educational Research Association, American Psychological Association, National Council on Measurement in Education. Standards for educational and psychological testing. 2014. https://www.testingstandards.net/uploads/7/6/6/4/76643089/standards_2014edition.pdf

[71] Kleinplatz PJ, Charest M, Paradis N, Rosen LA, Lawless S, Ramsay T. Remote Versus In-Person Group Therapy for Couples Distressed by Low/No Sexual Desire/Frequency or Sexual Desire Discrepancy: A Response to COVID-19. Journal of Couple & Relationship Therapy. 2024;24(1):111–31. doi: 10.1080/15332691.2024.2343730

[72] Trull TJ, Ebner-Priemer UW. Using experience sampling methods/ecological momentary assessment (ESM/EMA) in clinical assessment and clinical research: introduction to the special section. Psychol Assess. 2009;21(4):457–62. doi: 10.1037/a0017653 19947780 PMC4255457

[73] Mundfrom DJ, Shaw DG, Ke TL. Minimum Sample Size Recommendations for Conducting Factor Analyses. International Journal of Testing. 2005;5(2):159–68. doi: 10.1207/s15327574ijt0502_4

[74] Winton BG, Sabol MA. A multi-group analysis of convenience samples: free, cheap, friendly, and fancy sources. International Journal of Social Research Methodology. 2021;25(6):861–76. doi: 10.1080/13645579.2021.1961187

[75] Gosling SD, Mason W. Internet research in psychology. Annu Rev Psychol. 2015;66:877–902. doi: 10.1146/annurev-psych-010814-015321 25251483

[76] Pascoal PM, Shaughnessy K, Almeida MJ. A thematic analysis of a sample of partnered lesbian, gay, and bisexual people’s concepts of sexual satisfaction. Psychology & Sexuality. 2019;10(2):101–18. doi: 10.1080/19419899.2018.1555185

[77] Meade AW, Bauer DJ. Power and Precision in Confirmatory Factor Analytic Tests of Measurement Invariance. Structural Equation Modeling: A Multidisciplinary Journal. 2007;14(4):611–35. doi: 10.1080/10705510701575461

[78] Kenny DA, Kaniskan B, McCoach DB. The Performance of RMSEA in Models With Small Degrees of Freedom. Sociological Methods & Research. 2014;44(3):486–507. doi: 10.1177/0049124114543236

[79] Chen F, Curran PJ, Bollen KA, Kirby J, Paxton P. An Empirical Evaluation of the Use of Fixed Cutoff Points in RMSEA Test Statistic in Structural Equation Models. Sociol Methods Res. 2008;36(4):462–94. doi: 10.1177/0049124108314720 19756246 PMC2743032

