# Supplementary material for: Psychometric properties and qualitative evaluation of a Swedish translation of the New Sexual Satisfaction Scale–Short (NSSS-S)
Source: PLoS One. 2025 Aug 25;20(8):e0330353. doi: 10.1371/journal.pone.0330353 (PMC12377622; doi:10.1371/journal.pone.0330353)
Supplement: S1 File — (PDF) [file pone.0330353.s001.pdf]

S1 File. Swedish New Sexual Satisfaction Scale – Short form

Tänk på ditt sexliv under de senaste sex månaderna och skatta din tillfredsställelse med följande aspekter på en skala från 1 (Inte alls nöjd) till 5 (Extremt nöjd):

Om du har/har haft flera partners under de senaste sex månaderna, välj ut någon av dessa och svara utifrån denna. Om du inte har haft någon partner under de senaste sex månaderna, svara utifrån din senaste partner eller i relation till personer du är attraherad av i allmänhet.

	1 (Inte alls nöjd)	2 (Lite nöjd)	3 (Måttligt nöjd)	4 (Mycket nöjd)	5 (Extremt nöjd)
1. Kvaliteten i mina orgasmer					
2. Hur jag ”släpper taget” och hänger mig till sexuell njutning under sex					
3. På vilket sätt jag sexuellt reagerar på min partner					
4. Min kropps sexuella funktion					
5. Min sinnesstämning efter sexuell aktivitet					
6. Njutningen som jag ger min partner					
7. Balansen mellan vad jag ger och får vid sex					
8. Hur min partner känslomässigt öppnar upp under sex					
9. Min partners förmåga att få orgasm					
10. Min partners sexuella kreativitet					
11. Variationen av mina sexuella aktiviteter					
12. Frekvensen av min sexuella aktivitet					
