# Supplementary material for: Psychometric properties and qualitative evaluation of a Swedish translation of the New Sexual Satisfaction Scale–Short (NSSS-S)
Source: PLoS One. 2025 Aug 25;20(8):e0330353. doi: 10.1371/journal.pone.0330353 (PMC12377622; doi:10.1371/journal.pone.0330353)
Supplement: S2 File — (PDF) [file pone.0330353.s002.pdf]

S2 File. New Sexual Satisfaction Scale – Short form

Published in: Brouillard, P., Štulhofer, A., & Buško, V. (2019). The new sexual satisfaction scale and its short form. In R. R. Milhausen, J. K. Sakaluk, T. D. Fischer, C. M. Davis, & W. L. Yarbe (Eds.), *Handbook of Sexuality Related Measures* (4th ed., Issues 3–4, pp. 495–517). Routledge.

Thinking about your sex life during the last six months please rate your satisfaction with the follow aspects:

	Not at all satisfied	A little satisfied	Moderately satisfied	Very satisfied	Extremely satisfied
1. The quality of my orgasms					
2. My "letting go" and surrender to sexual pleasure during sex					
3. The way I sexually react to my partner					
4. My body's sexual functioning					
5. My mood after sexual activity					
6. The pleasure I provide to my partner					
7. The balance between what I give and receive in sex					
8. My partner's emotional opening up during sex					
9. My partner's ability to orgasm					
10. My partner's sexual creativity					
11. The variety of my sexual activities					
12. The frequency of my sexual activity					
